# The value of bedside teaching in undergraduate medical education: a literature review

**DOI:** 10.15694/mep.2020.000149.1

**Published:** 2020-07-22

**Authors:** Viswanathan Narayanan, Balakrishnan R Nair

**Affiliations:** 1 The University of Newcastle

**Keywords:** bedside, teaching, medical, education, physician, patient, student, perspectives, physical, examination

## Abstract

This article was migrated. The article was marked as recommended.

**Background:** Bedside teaching used to be an integral component of undergraduate medical education. In recent times, however, there has been a steady decline in the use of bedside teaching. This has occurred despite students, clinicians and patients viewing bedside teaching as valuable.

**Aims:** This review aims to appraise the current literature surrounding the perspectives in bedside teaching and evaluate its role within modern medical education.

**Methods:** A literature search was conducted in PubMed, Cochrane Library, and Ovid to identify appropriate studies. The journal articles were obtained by conducting sensitive and appropriate searches using keywords. All studies were examined comprehensively by the authors for suitability for inclusion.

**Results:** 2,770 records were identified from the initial search. An additional 3 records were identified after discussion with experts in the field. 583 duplicates were identified in the pool of records initially sourced. Of the remaining 2,190 records, 1,930 were excluded after inclusion and exclusion criteria were applied to their titles and abstracts. A further 252 records were excluded from the remaining 260 records after inclusion and exclusion criteria were applied to their full-texts. The remaining eight articles were reviewed by both authors and were deemed suitable for inclusion to the review.

**Conclusion:** The review showed that there is evidence in the literature to show that students, clinicians, and patients regard bedside teaching as beneficial. Discussions highlighted that bedside teaching can aid competency-based education models and cannot be replaced by simulation-based education. These results illustrate that, while there is evidence to show that bedside teaching holds value in medical education today, further studies should be conducted aiming to display long-term outcomes of bedside teaching.

## Introduction

Sir William Osler (1849-1919) commented: “To study the phenomena of disease without books is to sail an uncharted sea, while to study books without patients is not to go to sea at all” ( Stone, 1995). Osler is regarded as one of the most influential physicians and is still revered by the medical community worldwide. His teachings and principles are as relevant today as they were in his time. Decades ago, Osler had recognised the value of bedside teaching (BST) alongside traditional medical education. BST accounted for 75% of teaching time in 1964, it had regrettably declined to 16% by 1978 ( Ahmed, 2002). It can be reasonably assumed that this figure would be far lower today, in an environment saturated with medical technology such as advanced imaging ( Verghese, Shah and Harrington, 2018). As a medical community, we should not forget the value that BST can play in providing productive outcomes in medical education. This review is to examine the importance of BST within undergraduate medical education and explore the attitudes of students, patients and clinicians.

## Methods

To gather knowledge surrounding the literature of the topic in question, Viswanathan Narayanan conducted searches in PubMed, Cochrane Library, and Ovid, to identify original studies published between January 1960 to March 2020. To maximise the effectiveness of the search, keywords and their synonyms including “bedside teaching,” “perspectives, students, medical, clinicians, patients,” “competency, based, education, OSCE,” “simulation, teaching,” and “education, medical” were combined and included as keywords or MeSH terms. Only articles published in English were used. Articles with the perspectives of clinicians, students, and patients towards BST were identified using appropriate keywords. These keywords were used to identify articles that explore the relationships between simulation-based education (SBE)/competency-based education (CBE) and BST in undergraduate medical education. Each article was screened by its full title, and each abstract was examined for its applicability to this review. The reference lists of all the articles were reviewed to identify other potentially relevant studies. All articles were available in full text digitally.

## Results

The initial search of PubMed, Cochrane Library and Ovid returned 2,770 individual records. An additional 3 records were identified after discussion with experts in the field. Tables 1- 3 include depictions of the various search term combinations and syntax used, for each database. Tables 1- 3 include the number of individual results collected for each search.

**Table 1.** Search syntax used for PubMed

Database	Syntax	Number of results
PubMed(TIAB)	(Bedside teaching OR Bedside learning OR Bedside training) AND (Clinician perspective OR Clinician view OR Clinician attitude)	107
	(Bedside teaching OR Bedside learning OR Bedside training) AND (Medical student perspective OR Medical student view OR Medical student attitude)	149
	(Bedside teaching OR Bedside learning OR Bedside training) AND (Patient perspective OR Patient view OR Patient attitudes)	750
	(Simulation based education OR Simulation based learning OR Simulation based training) AND (Undergraduate medical education)	932
	(Competency based education OR Competency based learning OR Competency based training OR Competency based outcomes) AND (Bedside teaching OR Bedside learning OR Bedside training)	435
	(Osce OR Objective structured clinical examination*) AND (Bedside teaching OR Bedside learning OR Bedside training)	67

*AB* abstract, *TI* title

**Table 2.** Search syntax used for Cochrane Library

Database	Syntax	Number of results
Cochrane LibraryTI, AB, KW	(Bedside teaching OR Bedside learning OR Bedside training) AND (Clinician perspective OR Clinician view OR Clinician attitude)	3
	(Bedside teaching OR Bedside learning OR Bedside training) AND (Medical student perspective OR Medical student view OR Medical student attitude)	15
	(Bedside teaching OR Bedside learning OR Bedside training) AND (Patient perspective OR Patient view OR Patient attitudes)	41
	(Simulation based education OR Simulation based learning OR Simulation based training) AND (Undergraduate medical education)	172
	(Competency based education OR Competency based learning OR Competency based training OR Competency based outcomes) AND (Bedside teaching OR Bedside learning OR Bedside training)	35
	(Osce OR Objective structured clinical examination*) AND (Bedside teaching OR Bedside learning OR Bedside training)	14

*AB* abstract, *KW* keywords, *TI* title

**Table 3.** Search syntax used for Ovid

Database	Syntax	Number of results
OvidTI, AB, KW	(Bedside teaching OR Bedside learning OR Bedside training) AND (Clinician perspective OR Clinician view OR Clinician attitude)	0
	(Bedside teaching OR Bedside learning OR Bedside training) AND (Medical student perspective OR Medical student view OR Medical student attitude)	0
	(Bedside teaching OR Bedside learning OR Bedside training) AND (Patient perspective OR Patient view OR Patient attitudes)	1
	(Simulation based education OR Simulation based learning OR Simulation based training) AND (Undergraduate medical education)	17
	(Competency based education OR Competency based learning OR Competency based training OR Competency based outcomes) AND (Bedside teaching OR Bedside learning OR Bedside training)	4
	(Osce OR Objective structured clinical examination*) AND (Bedside teaching OR Bedside learning OR Bedside training)	28

*AB* abstract, *KW* keywords, *TI* title

From the pool of 2,773 records, 583 duplicates were identified. After duplicates were excluded, the remaining 2,190 unique records were screened with regards to title and abstract and if necessary in full text. Inclusion and exclusion criteria were applied to obtain articles relevant to the literature review. Table 4 outlines the inclusion and exclusion criteria that were utilised.

**Table 4.** Inclusion and exclusion criteria

Inclusion criteria	Exclusion criteria
-Articles discussing clinician perspectives of bedside teaching -Articles discussing medical student perspectives on bedside teaching -Articles discussing patient attitudes towards bedside teaching -Articles discussing the use of simulation -based education in undergraduate medical education -Articles comparing the use of simulation -based education with bedside teaching -Articles critically evaluating the use of simulation -based training techniques -Articles relating competency -based outcomes (e.g. OSCEs) with bedside training methods	-Non-English articles -No full text available through the University of Newcastle library -Articles that relate to advanced techniques (e.g. sigmoidoscopy) -Articles relating bedside teaching to highly specific educational outcomes (e.g. suturing skills) -Articles discussing the use of virtual reality training -Allied health related studies -Articles related to postgraduate medical education

**Figure 1.**
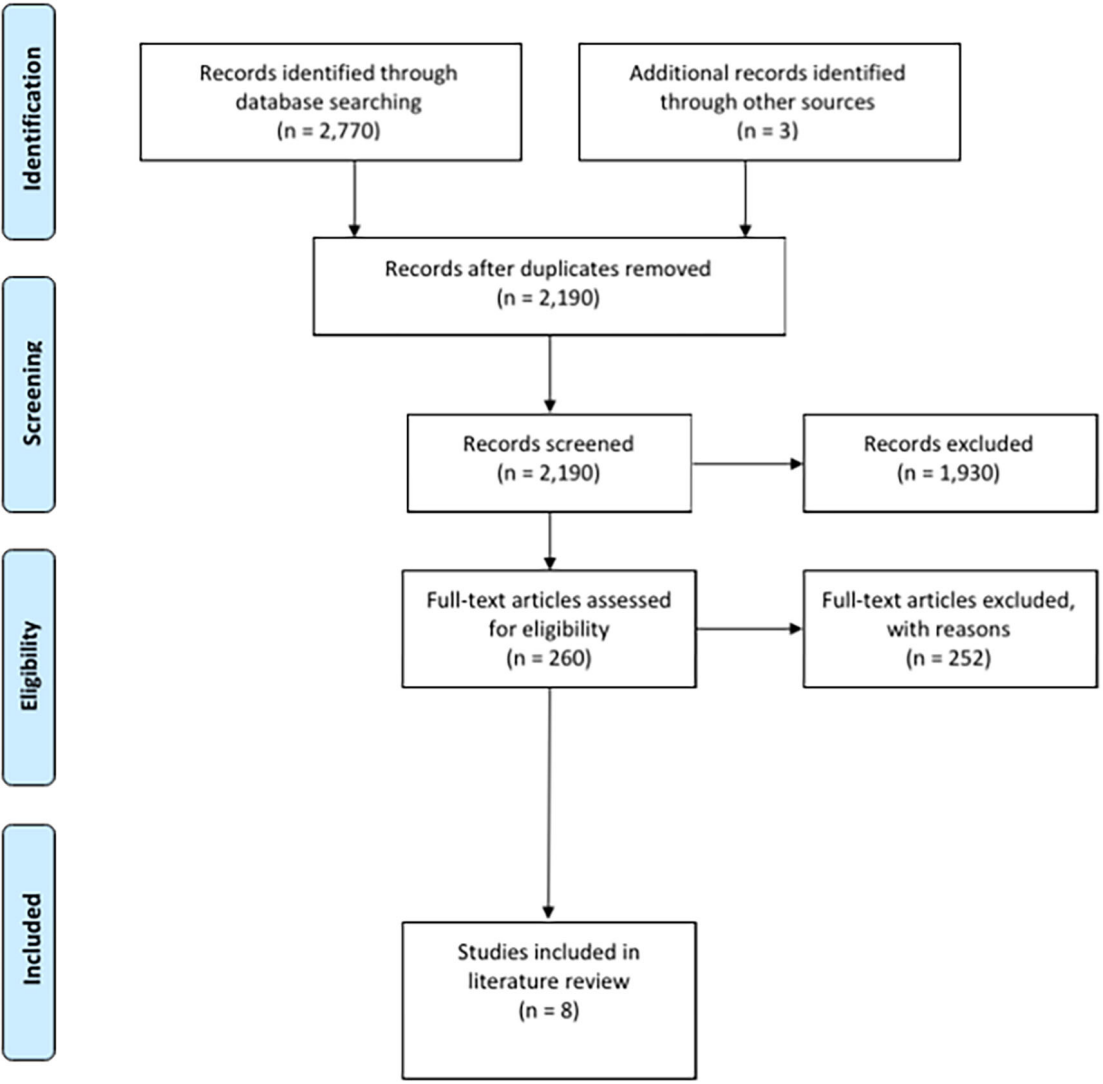
PRISMA flow diagram.

Adapted from Review Manager Web (RevMan Web).

1,930 records were excluded after screening the title and abstract of each record. Inclusion and exclusion criteria were applied comprehensively to the full text of the remaining 260 records. 252 records were excluded after screening the full text of each record. Both authors reviewed the final eight articles that remained and concluded that they contained useful information relating to patient perspectives of bedside teaching, student perspectives of bedside teaching, clinician perspectives of bedside teaching, simulation based education, and competency based education. The PRISMA checklist was utilised to facilitate the production of this review ( Moher *et al.*, 2009). A PRISMA flow diagram outlining the flow of information is illustrated in Figure 1.

## Discussion

The clinician’s perspective is paramount when discussing the utility of BST in undergraduate medical education. Ramani *et al.* (2003) identified a multitude of barriers for clinicians in providing BST to medical students. In particular, they noted a decline in BST skills, the perceived high expectations of BST quality, the lack of monetary value in teaching, and the erosion of BST ethics. These barriers highlighted areas of improvements needed for effective BST to be delivered to their intended recipients - medical students and other health professionals. Ramani *et al.* suggest that these barriers may be overcome through the provision of education and incentives to faculty. One of the limitations of this study is that it was conducted amongst a group of only 23 internists and subspecialists of a faculty in a single medical school. This small sample size significantly limits the generalisability of this study. Furthermore, this approach failed to capture the attitudes of clinicians in conducting BST in environments outside of the inpatient setting.

Furthermore, it can be of value to investigate how clinicians view the importance of BST and its place in the clinical setting. Tortez *et al.* (2016) conducted an anonymous survey administered to attendings (consultants) in six New York hospitals. In the 97 surveys that were returned, 92% of consultants found that bedside rounds are important for teaching, and 87% found it important for patient care. Seventy-seven per cent found that more emphasis on BST was needed and 71% reported that BST should be a priority. Contrary to these findings, Tortez *et al.* report that only 31% of consultants reported utilising BST. These results point to a dichotomous situation whereby consultants themselves identify that BST occupies a vital place in both learning and professional development, yet they nonetheless use BST sparingly. Although the evidence points to a clear awareness of the value of BST, consultants may face barriers, as explained earlier, in effectively using BST. This study highlights the need for support and education or otherwise for enabling clinicians to optimise the delivery of medical education. Therefore, further investigation is warranted to identify the most effective methods to reduce barriers inhibiting the provision of BST. However, societal and personal expectations may have resulted in bias in the responses received, which remains a limitation in this study. A possible solution, for example, could be to externally validate the self-reporting instrument by questioning how important the department, as a whole, felt towards BST.

To appreciate a holistic view of BST, we must also consider the perspectives of the recipient of BST, the medical student. One Australian multicentre study captured students’ opinion of BST ( Indraratna, Greenup and Yang, 2013). This study confirmed most students regard BST as being either extremely important or very important. Across all the disciplines, 90.4% of respondents in internal medicine claimed BST to be very important, while, on the lower end of the spectrum, 62.5% of respondents in psychiatry reported BST to be very important. The study also explored the perceived advantages and disadvantages of BST from students’ perspectives. Students found that the provision of feedback and the ability to appreciate clinical signs were the core benefits of BST. The negative aspects of BST were noted to be class sizes and time constraints. Importantly, this study demonstrated that students found BST to be a worthwhile endeavour as they self-report that BST had a positive impact on their education and learning. This study has been influential since it uncovered students’ underlying opinions regarding BST. Educators must, therefore, seek to fulfil the needs of students by providing high-quality BST. The method consisted of a self-reported survey, which may introduce bias. The study may have benefitted from far greater generalisability if there had been multicentre collaboration.

The final stakeholder of BST, the patient, must be included when evaluating the value of BST. Patients play a unique role within BST. Patients reap the benefits of BST by gaining medical knowledge alongside the student with increased transparency in their diagnosis and treatment. At the same time, they provide value to the student and the clinician by supplying indispensable information regarding their signs and symptoms. One study ( Nair, Coughlin and Hensley, 1997) examined the attitudes of students and patients regarding BST. Through a patient survey, they were successful in illustrating that 77% of patients enjoyed BST and 83% reported that it did not make them anxious. Furthermore, 68% reported that BST resulted in a better understanding of their illness. Only 7% of patients said BST was inappropriate, but they all clarified that use of medical jargon was a primary reason for this. In this study, BST is viewed positively from the patient’s perspective. This research shows the power of BST as a tool in the clinical environment. The study may have provided even greater insight if it had proceeded to investigate the quantitative outcomes of patients, for example, whether BST resulted in fewer complications during their hospital stay or adherence to medical advice. Added value could have been gained if characteristics such as the teaching methods used in the BST were explored further.

Simulation-based education (SBE) has become an integral part of the undergraduate medical curriculum in many countries worldwide. SBE aims to utilise simulation to recreate clinical scenarios in a variety of ways, ranging from full-body mannequins to simulated patients ( Al-Elq, 2010). Although there is evidence to show that SBE has a unique role to play in undergraduate education, it cannot replace traditional BST completely ( Bokken *et al.* 2008). There is a wide range of reported disadvantages associated with the use of SBE in medical education ( Krishnan, Keloth and Ubedulla, 2017). The most significant was discussed by Weller *et al.* (2012) as negative learning. Negative learning occurs due to defects in the design of the simulator or simulation. For example, if jaundice is not recreated by the simulator, an assumption may be reinforced that this is not a significant sign to notice during the physical examination. This same argument extends to a wide variety of other clinical signs that cannot be accurately replicated in a simulated scenario. Furthermore, with simulated scenarios, other core aspects of bedside practice, such as wearing personal protective equipment or gaining consent, may be omitted. Communication skills may also be developed artificially, rather than genuinely as a result of interaction with simulated patients. In addition, students may anticipate finding a clinical sign during SBE and would thus be in a state of hypervigilance; by contrast, BST may more effectively represent a clinical scenario. Other pitfalls cited include the cost and maintenance of high fidelity simulators. There is a paucity of data that definitively illustrates the effect SBE has on physical examination skills later in the careers of undergraduate students, and the field would greatly benefit from examining this area further. Although there is evidence for the use of SBE within medical education, the current evidence encourages the use of SBE as a supplement to traditional BST rather than as a substitute.

The intrinsic value of BST can also be found within competency-based undergraduate medical education. Competency-based education (CBE) is defined by Frank *et al.* (2010) as “[...]an approach to preparing physicians for practice that is fundamentally orientated to graduate outcome abilities and organized around competencies derived from an analysis of societal and patient needs.” Objective structured clinical examination (OSCE) is one such example of competencies that have been developed by medical schools in recent times to assess the competency of undergraduate medical students. In 2014, Roberts *et al.* (2014) developed a study to explore the effect of BST intervention on OSCE outcomes. In this study, there was a statistically significant difference in overall OSCE scores between third-year undergraduate medical students that undertook bedside physical examination training twice weekly and students that did not, over a period of 8 weeks. The 109 students that were subject to the intervention and the 85 controls had mean overall OSCE scores of 12.57 and 11.35 out of a possible score of 23, respectively. This study is significant since it provides evidence that BST can improve medical students’ physical examination skills and therefore increase their educational attainment within CBE models. This study demonstrates that BST can provide value concerning objective and quantitative undergraduate CBE outcomes. Furthermore, physicians widely report physical examination skills to hold considerable importance in hospital medical practice and, as such, BST must be promoted as a method of acquiring competency in physical examination amongst undergraduate students ( Elder *et al.* 2017). One of the limitations of this study, however, is that the providers of the BST were not given training before the intervention to standardise the education that students received in the intervention. This lack of training may have had an impact on students’ resulting OSCE scores. Furthermore, there is a chance that students may have witnessed the same cases during their ward rounds as they did in the OSCE. This potentially would distort the results from this study and act as a confounder. It may have been more useful for the educators to have been trained to provide the BST to the intervention group. Secondly, it may have been worthwhile to investigate the characteristics of the BST to which students were subjected, for example, the illnesses they observed, the nature of the teaching, and how frequently they were asked to elicit signs during BST.

Notwithstanding this study, the literature still suffers from a dearth of studies wherein BST is assessed with respect to quantitative student outcomes. We notice a variety of ways in which students are quantitatively assessed during medical school. These include OSCEs, written examinations, evaluations, and assignments, all of which form constituents of CBE. Devising further studies that aim to correlate the use of BST with such quantitative outcomes would add strength to the argument supporting increased use of BST in the education of medical students.

## Conclusion

BST has declined as a component of undergraduate medical education over recent decades. However, there is evidence to show that BST can benefit all three parties involved: the student, the patient, and the clinicians. Despite the advent of technology in the clinical environment, the value that BST provides for the next generation of physicians cannot be overstated. Considering that 56% of patient problems are correctly diagnosed at the end of medical history-taking and that this figure rises to 73% by the end of the physical examination, BST remains as useful today as it did when medicine began ( Sandler, 1980). Therefore it would be short-sighted to underestimate the value of BST in educating future physicians. As Flexner said in 1925, “The facts are locked up in the patient. To the patient, therefore, [the student] must go” ( Huth and Murray, 2006).

## Take Home Messages

• The usage of bedside teaching in undergraduate medical education has declined over recent decades.
• Evidence shows that bedside teaching is still perceived as valuable by students, patients, and clinicians.
• Bedside teaching has an important role to play in undergraduate education and cannot be replaced by simulation-based education.
• Further studies are warranted to focus on presenting the long-term benefits of bedside teaching.

## Notes On Contributors

Viswanathan Narayanan

Viswanathan Narayanan is a final year medical student at the University of Newcastle, Newcastle, Australia. ORCiD: https://orcid.org/0000-0003-4019-1692

Professor Balakrishnan R Nair AM

MBBS, MD (Newcastle) FRACP, FRCPE (Edinburgh), FRCPG (Glasgow), FRCPI (Ireland), FANZSGM, Graduate Dip (ClinEpid)

Professor Balakrishnan R Nair is the Professor of Medicine and Associate Dean, School of Medicine and Public Health, University of Newcastle, Australia. He holds the position of Director of the Centre for Medical Professional Development, Hunter New England Health Service at Newcastle. He is assigned to the Health Education & Training Institute of NSW as the District Medical Director. ORCiD: https://orcid.org/0000-0002-9100-4298
