# Supplementary material for: The value of bedside teaching in undergraduate medical education: a literature review
Source: MedEdPublish (2016). 2020 Jul 22;9:149. [Version 1] doi: 10.15694/mep.2020.000149.1 (PMC10702672; doi:10.15694/mep.2020.000149.1)
Supplement: Peer review history

# Reviewer report: Reviewer response for version 1

**DOI:** 10.21956/mep.20039.r30674

**Published:** 2020-08-09

**Authors:** Subha Ramani

**Affiliations:** 1 Harvard Medical School, Brigham and Women's Hospital

This review has been migrated. The reviewer awarded 4 stars out of 5 It is very nice to read a paper written by a student guided by a seasoned clinician. In general, this is the sort of mentoring we should be actively encouraging in scholarship. The topic is also important and I agree with the authors that for the last nearly 2 decades bedside teaching has been declining and yet we do not seem to have a good handle on this challenge. I agree also that bedside teaching is more than inpatient rounds, it is wherever patients are present and the discussion engages around clinical skills and patient care.More recent models focus on being time-efficient, but it is not clear how well the tenets of good bedside teaching are being followed. Just being present at the bedside does not mean that quality bedside teaching is happening. I am seeing increasing frequency of 'bedside' rounds where one might think that the computer is the patient.The pandemic only makes teaching and learning at the bedside more challenging and I fear that clinical skills will become an anachronism.Finally, I concur with the authors that more bedside teaching research needs to happen from the patient perspective. Though there are a few studies, we need more.This paper has made an effort to review existing literature and summarize challenges and recommendations. There are several more papers that have not been cited- Williams et al, Fletcher at al, Gonzalo et al. etc. I would see this paper as a start, more studies need to be done to actually stimulate more teaching in the presence of patients and do it well, especially engaging the patient and making an impact on patient care.My star rating is based upon the efforts by the student and his mentor to bring the topic of bedside teaching back to the forefront and I hope they will conduct more research on this topic and move the needle forward.

Reviewer Expertise:

NA

No decision status is available

# Reviewer report: Reviewer response for version 1

**DOI:** 10.21956/mep.20039.r30672

**Published:** 2020-08-06

**Authors:** Michael SH Wan

**Affiliations:** 1 University of Notre Dame

This review has been migrated. The reviewer awarded 3 stars out of 5 Really good to see medical students interested in Medical Education research and this is a good start. The paper focused on an important area in learning and teaching of recognition of proper clinical signs and clinical reasoning via BST. We were taught that the best way to learn is from the patients and in the wards working with health professional team. The aims and research questions could be stated more explicitly. The trend towards less BST and the shift towards simulation and online learning is important to be recognised. Although simulation could support learning in a safe environment especially in critical/deteriorating patient scenarios and improve communication skills among health professionals (interprofessional learning), this could not replace BST entirely. In the discussion, some suggestions on how to 'promote' BST in the teaching community would be helpful.

Reviewer Expertise:

NA

No decision status is available

# Reviewer report: Reviewer response for version 1

**DOI:** 10.21956/mep.20039.r30676

**Published:** 2020-08-04

**Authors:** Ian Wilson

**Affiliations:** 1 International Medical University

This review has been migrated. The reviewer awarded 3 stars out of 5 This is an interesting and important review particularly at the current time when there are restrictions on the amount of BST possible because of the pandemic. the paper is good start but I wish it had provided more details on numbers of participants in each study. with eight papers covering students, doctors and patients it is unclear how big the studies are.Overall it is an interesting paper that should stimulate more research. It would be good to see the author to extend this into a research project.

Reviewer Expertise:

NA

No decision status is available

# Reviewer report: Reviewer response for version 1

**DOI:** 10.21956/mep.20039.r30673

**Published:** 2020-07-26

**Authors:** Judy McKimm

**Affiliations:** 1 Swansea University

This review has been migrated. The reviewer awarded 3 stars out of 5 It is great to see a medical student engaging on educational research, supported by faculty. I thought this posed interesting issues. I think it could have been improved in a few ways however, which is meant as constructive criticism:(1) a clearer research question or sub question would have focussed the paper more clearly(2) the paper needed putting into more context, for example, a definition or more of 'bedside teaching', does this include board rounds, simulation, ambulatory settings, theatres etc? or is it simply teaching/learning (again does this include unsupervised interaction with patients?) in an in-patient ward setting? does it include ward rounds (teaching or business?) etc etc(3) following these points up would then have sown the seeds for a more comprehensive discussion and conclusions/recommendations as to the benefits, barriers, types etc of BST - building on the comments from Prof Wilkinson belowOverall this was a good start, I think it provides a basis for a review of this paper and also more research in this area around the changing face of BST and when and how it should be preserved as well as when other modalities (such as simulation) can be used

Reviewer Expertise:

NA

No decision status is available

# Reviewer report: Reviewer response for version 1

**DOI:** 10.21956/mep.20039.r30677

**Published:** 2020-07-25

**Authors:** Tim Wilkinson

**Affiliations:** 1 University of Otago,Christchurch

This review has been migrated. The reviewer awarded 3 stars out of 5 Nice summary of the literature, led by a final year medical student. The research question wasn’t that clear but I think was to summarise the place of BST from the perspectives of teacher, learner and patient. A second RQ may have been to compare BST with simulation. Maybe the RQ could be stated more clearly earlier on?I wonder whether outpatient teaching with a patient should also have been included, given (hopefully) fewer patients are stuck in beds these days?I thought the results section ended a bit abruptly with just the outcomes of the search. A summary of the types of studies and their key findings would fit better at the end of the results and then the discussion can focus more on the synthesis and meaning of what was found. Arguably the inclusion and exclusion criteria would be better in the methods section.This paper made me think that the next step might be to build on it with a realist review looking at why BST is effective and/or it what circumstances is it most effective. The authors also raise the question of how barriers could be removed

Reviewer Expertise:

NA

No decision status is available

# Reviewer report: Reviewer response for version 1

**DOI:** 10.21956/mep.20039.r30675

**Published:** 2020-07-24

**Authors:** Chris Roberts

**Affiliations:** 1 OPME - University of Sydney NSW AUSTRALIA

This review has been migrated. The reviewer awarded 4 stars out of 5 It is always good to see medicals students involved in medical education research. COVIID 19 impacts have left many medical students without access to regular bedside teaching. At the same time there has been a flight by educators into online teaching modalities. It is very timely that there is an updating review on the value of bedside teaching (BST). BST is important in medical education for practicing history taking, clinical examination and differential and management. This study examine the importance of BST within undergraduate medical education and explores the attitudes of students, patients and clinicians. There is a secondary aim to compare the BST with what has often been used to emulate BST simulation based learning. It is surprising that so few papers can be found which define the impact of BST on learning outcomes. Yet the authors had broad search terms, used standard techniques of the usual databases and screened many potential articles. It seems that student like BST patient are happy to be partners in BST, but there is need for major faculty development to re-engage clinical teachers. Overall this papers call for more studies seems an important one wherein BST is investigated with respect to quantitative student outcomes. It seems we have taken BST for granted.

Reviewer Expertise:

NA

No decision status is available
